# Mental health in Eritrea: A brief overview and possible steps forward

**DOI:** 10.7189/jogh.11.03018

**Published:** 2021-01-16

**Authors:** Fikrejesus Amahazion

**Affiliations:** College of Arts and Social Sciences, Adi Keih, Eritrea

Mental health is an integral component of health. Recently, the global burden of mental disorders has grown, with significant impacts on health and major social, human rights, and economic consequences [1,2]. Despite its significance, however, mental health has historically been neglected on Africa’s health and development policy agenda, while there has been little research into mental health problems [3]. Across Africa, health systems have not adequately responded to the burden of mental disorders, and a large treatment gap prevails [4-6]. There is also widespread ignorance about the extent of mental health problems and a prevalence of stigma and discrimination toward those with mental illness [7,8].

In Eritrea, a developing country located in Northeast Africa, mental health is a rising problem (**Table 1**). Since winning independence in 1991, Eritrea has made considerable progress within the health sector, including in life expectancy, maternal, infant, and child mortality, immunization, and control of major diseases [9-12]. However, the country also faces significant challenges, including regional conflict, poverty, socio-political challenges, erratic rainfall and potential for drought, food security, and shortage of skilled labor [9-12]. Additionally, mental health is a growing issue, although there have been few studies on the topic.

**Table 1:** Eritrea mental health statistics

Population	3 500 000
GDP per capita	US$780
Patient-Doctor ratio	0.5:10 000
Mental disorder prevalence	14.5%
Children with intellectual disabilities	30-40 000
Burden associated with mental disorders (disability-adjusted life-years)	2704.61 per 100 000 population
Suicide mortality rate	7.9 per 100 000 population
Mental hospital beds	4.13 per 100 000 population
Mental health workers	2.48 per 100 000 population

## GENERAL HEALTHCARE STRUCTURE

Eritrea’s national health policy aims to ensure equity and access to health services at an affordable cost. Priorities include addressing maternal and child health, as well as controlling communicable diseases. Health services are delivered utilizing a referral system based on three tiers, comprising primary-level facilities (health stations and health centres), secondary-level facilities (first contact or sub-zone hospitals and zonal referral hospitals) and tertiary-level facilities (national referral hospitals). While there are no private health facilities in the country, there is a system of private practice within government health facilities by way of a partnership between the Government and health workers [12-14].

**Figure:**
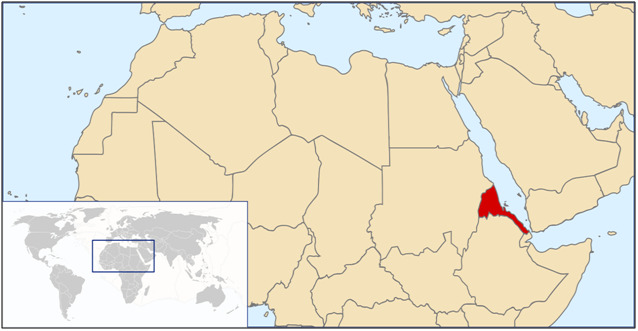
Photo: Eritrea geographic setting (Image credit: https://commons.wikimedia.org/wiki/File:LocationEritrea.svg).

Eritrea faces numerous challenges within health care. It has deficits in financing, access to basic technologies, medicines, and medical information systems, while lacking a skilled health workforce necessary for combating noncommunicable diseases (NCDs). Furthermore, health care workers often have a heavy workload related to a low doctor to patient ratio (approximately 0.5 per 10 000 people).

## MENTAL HEALTH IN ERITREA

There is a rising frequency of mental health problems in Eritrea, and mental illnesses are among the leading causes of disability, comorbidity, and mortality. According to a 2014 local mental health survey, Eritrea has a common mental disorder prevalence rate of about 14.5% [15]. In 2015, there were an estimated 219 549 cases of depressive disorders (4.3% of the population) and 156 599 cases of anxiety disorders (3.1%) [16]. Other common conditions in Eritrea include schizophrenia, bipolar affective disorder, neurotic, stress, and mood disorders, epilepsy, and dementia. Notably, approximately 30-40 000 children are estimated to have intellectual disabilities.

Mental disorders lead to considerable losses in health and functioning in Eritrea. The burden associated with mental disorders in disability-adjusted life-years is approximately 2704.61 per 100 000 population. The burden of depressive disorders is about 40 426 years lived with disability (constituting 8.2% of total years lived with disability), while the burden of anxiety disorders is 14 474 years lived with disability (2.9%). Mental disorders also contribute to premature mortality; the suicide mortality rate (per 100 000 population) in Eritrea is about 7.9 [16-18]. Due to human resource constraints and a lack of specialized skills, it is probable that many mental illnesses and disorders are undetected or misdiagnosed.

Eritrea is characterized by several socio-political and economic factors associated with mental illness. Due to war, regional instability, and displacement, many Eritreans have suffered heavily from depression and posttraumatic stress disorder. Other significant issues include poverty, unemployment and underemployment, and single-headed households.

## CARING FOR THE MENTALLY ILL

Psychiatric services were first introduced in Eritrea during the period of Italian colonization. Today, the country has a limited network of mental health facilities and services. While Eritrea has a national mental health policy, it does not have mental health legislation. Moreover, although current data are unavailable, like most low-income countries mental health has traditionally comprised a small proportion of the national health budget. This is largely due to the fact that the country has prioritized public health fundamentals, such as clean water, hunger, sanitation, and communicable diseases.

The country’s sole mental hospital, St. Mary’s Neuro-Psychiatric Hospital, provides inpatient care and long-stay residential services. Built in 1971 and located in Asmara, the capital, it is equipped with several hundred beds and also offers outpatient services. As well, there is a community residential care facility which serves those with relatively stable and chronic mental disorders not requiring intensive medical interventions. This community-based facility is also located in Asmara and hosts several hundred beds. Nationally, there are about 4.13 mental hospital beds per 100 000 population [17]. The majority of spending on mental health goes to St. Mary’s Neuro-Psychiatric Hospital, which serves only a small proportion of those needing care.

In addition, there are 10 psychiatric units attached to general hospitals, while all six administrative zones of the country have at least one psychiatric nurse. In 2017, about 23% of the country’s secondary and tertiary level health care facilities provided outpatient or inpatient mental health services.

There is a high disparity in urban-rural distribution of mental health workers and services, with most located in urban areas. Consequently, patients from rural areas have reduced accessibility to services. Another constraint is scarcity of mental health professionals. There are about 2.48 mental health workers per 100 000 population. Additionally, Eritrea has approximately 0.08 psychologists, 0.04 social workers, and 2.17 mental health nurses per 100 000 population. There is one psychiatrist in the country, although several doctors have been sent abroad for training in psychiatry. Generally, these figures all fall below global averages [17].

Essential psychotropic medicines are available within many health facilities. However, distribution can often be limited, especially with geographical distance from health care providers negatively impacting accessibility in some areas. Patients do not pay for psychotropic medicines (or care and services), which is important since many patients are poor. Additionally, some individuals with mental illnesses receive medications and other support from relatives abroad.

Families and caregivers frequently play an important role in the care of the mentally ill. However, many lack the resources or specialized skills to provide proper care. Furthermore, the added responsibilities and costs associated with care can lead caregivers to experience significant physical or emotional stress, employment or financial challenges, and health problems.

## ALTERNATIVE TREATMENT

A large proportion of people with mental illness consult alternative practitioners for mental health treatment. With Eritrea being a traditional, conservative, religion-bound society, many people with mental illness (or their families) turn to traditional healers or religious leaders for treatment as they are perceived as culturally appropriate. Moreover, traditional healers or religious advisers are often more geographically accessible; Eritrea is a predominantly agricultural country and most health facilities and services are concentrated in urban areas. Although alternative practitioners can provide important support, very few traditional or alternative treatment practices have sound empirical databases to suggest their effectiveness and safety. Furthermore, initial consultation with alternative practitioners may delay access to care and exacerbate the course or outcome of illnesses.

## STIGMA AND DISCRIMINATION

Mental illness in Eritrea is stigmatized and poorly understood. It is often viewed as a punishment from God, the result of witchcraft, sorcery, or possession by evil spirits (eg, the “evil eye”), contagious, and a source of great shame. Moreover, people with mental illness often encounter isolation or exclusion, experience ridicule or blame, and are frequently considered dangerous, dependent, and unfit for marriage or work. It is not uncommon for family members of mentally ill persons to also experience stigma themselves.

Stigma often serves as a barrier to treatment, inhibiting both the mentally ill and their families from acknowledging the condition or seeking help. For example, many patients and their families are often concerned or afraid about how other people will view or treat them if knowledge of their condition becomes public, thus reducing the likelihood of seeking treatment.

There are legislative measures in place to help rectify discrimination and ensure equality of opportunity, access, and participation in all aspects of life for people with mental illnesses and disabilities. These include legal articles on discrimination related to employment, termination or dismissal, and wages, as well as social support for state housing and subsidized housing.

## MOVING FORWARD

### More information and awareness

As health and living standards improve, concomitant with significant reductions in communicable and preventable diseases, there is a gradual shift in burden toward NCDs in Eritrea. Mental illnesses and disorders also will likely continue to grow as a challenge, in terms of prevalence, cause of disability, and contribution to overall burden of disease. More and methodologically rigorous population-based studies and data collection are necessary to estimate the prevalence, burden, and impact of mental illness and provide relevant information for government, policymakers, and health care professionals.

There is a considerable need to increase public awareness about mental health and reduce stigma. Efforts may be linked to existing government and community-based programs focused on general health and rights issues. Moreover, these campaigns can involve public workshops and seminars or utilize local media. Since Eritrea is ethno-linguistically diverse, educational and awareness materials should be made available in all languages used within the country, while various communities, elders, and other respected figures should be involved in design and dissemination.

### Improved services and professional development

The scarcity of mental health professionals remains a major challenge. More mental health specialists, especially psychiatrists and psychologists, should be trained. Increasing psychotherapy in the country can also help reduce disability, morbidity, and mortality, improve functioning, and decrease hospitalization. Partnerships should be established with foreign programs to provide more scholarships, fellowships, and other opportunities. International experts and organizations can support the development and expansion of local mental health-related educational departments.

Additionally, mental health services should be decentralized and integrated into general primary and secondary health services to improve access to services and reduce the treatment gap. With primary health care the first point of contact between people and health services, integration can achieve early identification of mental disorders and promote good mental health through direct provision and referrals to more specialized services. The experiences of mental health systems in other countries with similar socio-cultural or economic contexts can be explored to identify lessons. Importantly, for a low-income country like Eritrea, the correct diagnosis and treatment of patients with mental disorders at primary care levels can lead to considerable cost savings as well as a reduction in the workload of primary care workers. It can also help reduce indirect costs for patients associated with seeking specialist care in distant locations.

This will require a task-sharing or task-shifting approach, involving the use of non-specialist health care providers. Specifically, primary care physicians, nurses, and general health professionals should be trained in the necessary skills to screen, diagnose, and manage common mental health conditions or to refer patients for specialized care. One cost-effective option is the World Health Organization’s Mental Health Gap Action Programme, which can be used to build capacity among non-specialist health care providers in assessment and management of people with mental illnesses. Experts and trainers from international organizations can be consulted during the planning and implementation of these programs. As well, alternative practitioners, such as traditional healers and religious leaders (who are knowledgeable about cultural norms and possess significant social capital and local legitimacy), can be integrated into the treatment process through outreach and training in order to reach more people suffering from mental disorders.

The availability and distribution of psychotropic medicines, which are an essential component of mental health care, must be improved, especially in remote and rural areas. This can be encouraged through ensuring the availability of essential medicines at all levels of the health care system and training nurses and non-specialist health care providers in appropriate prescription and dispensation. Notably, more emphasis on developing or improving psychotherapeutic skills in general health care workers can help ease psychic pain in people unable to reach psychiatric service.

### Continued high-level commitment

Improvement of mental health care and services requires continued high-level political commitment and leadership. Although the existence of a mental health policy is positive and encouraging, the government should ratify the *United Nations Convention on the Rights of Persons with Disabilities* (UNCRPD) and also enact progressive mental health legislation inline with international norms and standards. Ratification of the UNCRPD can demonstrate high-level prioritization of mental health, increase awareness, promote the rights of people with mental illnesses, and help change negative attitudes and beliefs. Furthermore, legislation can provide a regulatory framework for addressing critical mental health issues and help ensure those suffering from mental illnesses are afforded protection and receive improved treatment and care.

## CONCLUSION

Mental health is a growing challenge in Eritrea. With the issue not having received great attention to date, there are numerous possibilities for future research. For instance, work may explore significant factors or consequences associated with various disorders. As well, future work can examine effectiveness of different interventions and therapies, or investigate potential social and cultural barriers.
